# The Association between Serum Vitamin D Levels and *Helicobacter pylori* Presence and Eradication

**DOI:** 10.3390/nu13010278

**Published:** 2021-01-19

**Authors:** Asher Shafrir, Michal Shauly-Aharonov, Lior H. Katz, Ora Paltiel, Yishai Pickman, Zvi Ackerman

**Affiliations:** 1 Division of Medicine, Meuhedet Health Services, Tel Aviv 6203854, Israel; 2 Hadassah Medical Center, The Department of Gastroenterology, Faculty of Medicine, Ein Karem Campus, Hebrew University of Jerusalem, P.O. Box 12249, Jerusalem 9112102, Israel; klior@hadassah.org.il; 3 The Jerusalem College of Technology, P.O. Box 16031, Jerusalem 91160, Israel; michal.shauly@mail.huji.ac.il; 4 Braun School of Public Health and Community Medicine, Hebrew University of Jerusalem, Jerusalem 9112102, Israel; Orap@hadassah.org.il; 5 Hadassah Medical Center, Department of Hematology, Faculty of Medicine, Ein Karem Campus, Hebrew University of Jerusalem, Jerusalem 9112102, Israel; 6 K-Health Inc., Tel Aviv 6581706, Israel; p.Yishai@gmail.com; 7 Hadassah Medical Center, Department of Medicine, Faculty of Medicine, Mount Scopus Campus, Hebrew University of Jerusalem, P.O. Box 24035, Jerusalem 91240, Israel; zackerman@hadassah.org.il

**Keywords:** *Helicobacter pylori* infection, *Helicobacter pylori* eradication, vitamin D, 25-hydroxyvitamin D2, Israel

## Abstract

Background: The success of *Helicobacter pylori* (*H. pylori*) eradication depends on several host and treatment factors. Serum vitamin D levels may be associated with *H. pylori* infection and eradication rates. We investigated the association between vitamin D and *H. pylori* infection and eradication, using a large electronic database based on medical records from a population-based health maintenance organization. Methods: Data regarding adults who underwent *H. pylori* testing and had vitamin D measurements within one month of *H. pylori* testing were collected. *H. pylori* infection was ascertained using urea breath or stool antigen tests. A negative *H. pylori* test following a positive result implied eradication. Multivariate regression models were constructed to assess associations between *H. pylori* infection, eradication, and vitamin D. Results: Among 150,483 members who underwent *H. pylori* testing from 2009 to 2018, 27,077 (18%) had vitamin D measurements. Vitamin D levels were inversely associated with *H. pylori* infection, *p* < 0.001. The odds of a positive *H. pylori* test were 31% higher among patients with vitamin D levels <20 ng/mL, compared with those with levels ≥20 ng/mL (OR 1.31, 99% CI 1.22–1.4, *p* < 0.001). Purchase of vitamin D supplements was associated with a negative subsequent *H. pylori* test (*p* < 0.001). Mean vitamin D levels were moderately higher in those with successful vs. failed *H. pylori* eradication (19.34 ± 9.55 vs. 18.64 ± 9.61, *p* < 0.001). Conclusions: Vitamin D levels are associated with *H. pylori* infection. Increased vitamin D levels are associated with successful *H. pylori* eradication. Vitamin D may have a role in *H. pylori* eradication.

## 1. Introduction

*Helicobacter pylori* (*H. pylori*) is a gram negative bacterium that colonizes the human gastric mucosa [1,2]. Multiple studies have shown that *H. pylori* infection is associated with chronic gastritis, peptic ulcer disease, mucosa-associated lymphoid tissue lymphoma, and gastric cancer [1,2]. Eradication of *H. pylori* has been shown to decrease the incidence of gastric cancer among patients with a family history of gastric cancer [1,2]. The presence of seasonal variation in the frequency of upper gastrointestinal bleeding due to peptic ulcer disease, with rates higher in the winter and spring and lower in the summer, was reported [3]. Several hypotheses were raised to explain the seasonal variation in the clinical presentation and detection of *H. pylori* infection. These include seasonal changes in the immune system, nutritional status, and medication use [4]. Infection rates of *H. pylori* may also depend on environmental factors, such as altitude, average annual temperature, and average daily sunshine [5], factors which may influence ultraviolet light intensity and exposure time and the synthesis of vitamin D [6].

*H. pylori* eradication is indicated for any patient found to be infected with the organism [7]. Many patients fail first line treatment and require further lines of antibiotic treatments, of which several options exist [8]. Recently, observational studies have described various associations between *H. pylori* infection and serum vitamin D levels [9,10,11,12,13,14,15,16,17]. These publications are based on retrospectively collected data from small heterogeneous groups of patients from different parts of the world. In these studies, serum vitamin D levels were reported, without mentioning the calendar period in which they were measured. It is well known that serum vitamin D levels vary according to season, being higher in the hot and sunny seasons [18]. In the present study, we assessed associations between serum vitamin D and *H. pylori* infection, and between vitamin D levels and supplementation and *H. pylori* eradication, using data extracted from electronic medical records (EMRs) of members insured by a large Israeli Health Maintenance Organization (HMO).

## 2. Materials and Methods

Meuhedet HMO is the third largest healthcare provider in Israel. The HMO serves over 1,200,000 individuals and is generally representative of the general population of Israel. Meuhedet’s comprehensive and integrated computerized database includes real time input from all physician visits, medical diagnoses, laboratory results, hospitalizations, and dispensing data on prescription medications and nonprescription over-the-counter (OTC) medications. Data from EMRs for all insured individuals aged 18 and over who underwent a urea breath test (UBT) or *H. pylori* stool antigen tests between 1 January 2009–31 December 2018 were extracted. Patients were categorized as *H. pylori* positive or negative based on the results of their first test, according to the accepted normal values of the UBT and stool antigen tests.

In Israel, performing an UBT or a stool antigen test to verify *H. pylori* eradication, after the completion of an antibiotic course against *H. pylori*, is an accepted and a regularly performed medical practice. As such, any subsequent test for the presence of *H. pylori* infection that was performed following a prior positive result, without a specific time limit, was considered as a test done for the purpose of verifying *H. pylori* eradication.

Additional data extracted from the EMRs included the following: patient’s age, gender, body mass index (BMI), and date of *H. pylori* testing. Vitamin D levels performed within a 30-day period prior to or after *H. pylori* testing were included in our dataset, along with blood counts, liver and kidney function tests, cholesterol, ferritin, vitamin B12, C-reactive protein, glycosylated hemoglobin, and thyroid stimulating hormone levels. In addition, all medical diagnoses documented at any point prior to *H. pylori* testing (e.g., hypertension, diabetes mellitus, hyperlipidemia, and hypothyroidism) were recorded.

In order to minimize false negative UBT results, a sub-group of subjects who were not prescribed any proton pump inhibitors (PPI) in the three months prior to the *H. pylori* test was identified and a sub-analysis was performed in this group. Data regarding prescriptions used for *H. pylori* eradication were also available. Eradication protocols were prescribed mainly by family physicians at their professional discretion and included: (a) bismuth-based quadruple therapy (bismuth, metronidazole, tetracycline, and PPI), (b) clarithromycin-based triple therapy (clarithromycin, either amoxicillin or metronidazole and PPI), (c) clarithromycin-based quadruple therapy (clarithromycin, amoxicillin, either metronidazole or tinidazole and PPI), or (d) triple therapy (either PPI or bismuth, and two of the following antibiotics: amoxicillin, metronidazole, and tetracycline).

In Israel, vitamin D supplements can be purchased either as a prescription medication or OTC. In order to investigate whether there is any association between taking vitamin D supplements and *H. pylori* infection, we searched EMRs for records regarding purchase of vitamin D in the three months prior to the *H. pylori* test. Serum vitamin D (25(OH)D) levels were measured at a single central laboratory using the Architect^®^ 25-OH vitamin D assay (Abbott Diagnostics, Chicago, IL, USA) [19].

This research was conducted in accordance with the Declaration of Helsinki and approved by the research ethics committee (IRB) of Meuhedet HMO (01-26-06-19). The author and all co-authors had access to the study data and reviewed and approved the final manuscript.

### Statistical Analysis

Statistical analyses were performed using R software (R Development Core Team, 2018). Univariate analysis was performed using Chi-squared test to compare categorical variables, and *t*-test to compare means of (1) vitamin D levels in patients positive and negative for *H. pylori*, and (2) vitamin D levels in patients with successful and unsuccessful eradication.

Logistic regression was performed to assess the effect of vitamin D level on *H. pylori* infection, controlling for patient characteristics that had been found in the literature to be potential confounders. Variables with a *p*-value less than 0.2 on univariate analysis were considered as potential confounders (and thus were included in the covariate selection process in the multivariate analysis). These included gender, age, hypertension, diabetes mellitus, hypothyroidism, ischemic heart disease, and creatinine levels. To avoid collinearity, separate logistic models were constructed to evaluate the association between purchase of vitamin D supplementation and *H. pylori* infection. Another logistic analysis was performed to estimate the effect of vitamin D on success in *H. pylori* eradication. This model included gender, age, and receiving appropriate treatment, as potential confounders.

Due to the large sample size, *p*-values of less than 0.01 were considered statistically significant in all analyses; choosing this value for significance level also addressed the issue of multiple comparisons. Since *p*-values are often small in studies with large sample sizes, it is more meaningful to consider the effect size. To this end, for descriptive statistics, the effect size is measured by differences of means (or percentages) normalized by the estimate of a standard deviation. In the logistic regressions, odds ratios and their corresponding 99% confidence intervals are presented.

## 3. Results

A total of 258,626 tests were performed for the presence of *H. pylori* infection between 1 January 2009 and 31 December 2018. Of these, 150,483 were considered primary tests. UBT comprised 99% and the rest were stool antigen tests. Approximately 50% of those tested (*n* = 75,640) were found to be *H. pylori* positive. The mean age of the *H. pylori* negative subjects was higher than that of the *H. pylori* positive subjects (42.15 ± 16.65 vs. 40.95 ± 14.72 years, *p*-value < 0.001). Women were slightly more likely to be *H. pylori* negative than men (50% vs. 49%, respectively, *p*-value < 0.001). *H. pylori* negative individuals were more likely than *H. pylori* positive subjects to suffer from various comorbidities, such as type 2 diabetes mellitus (6.97% vs. 5.89%, *p*-value < 0.001), hypertension (8.35% vs. 6.14, *p*-value < 0.001), ischemic heart disease (4.33% vs. 3.0%, *p*-value < 0.001), hypothyroidism (8.67% vs. 6.56%, *p*-value < 0.001), and fatty liver disease (4.63% vs. 3.52%, *p*-value < 0.001). Additional comparisons are presented in Table 1. Although the differences tend to be statistically significant, the effect sizes tended to be quite small.

**Figure 1:**
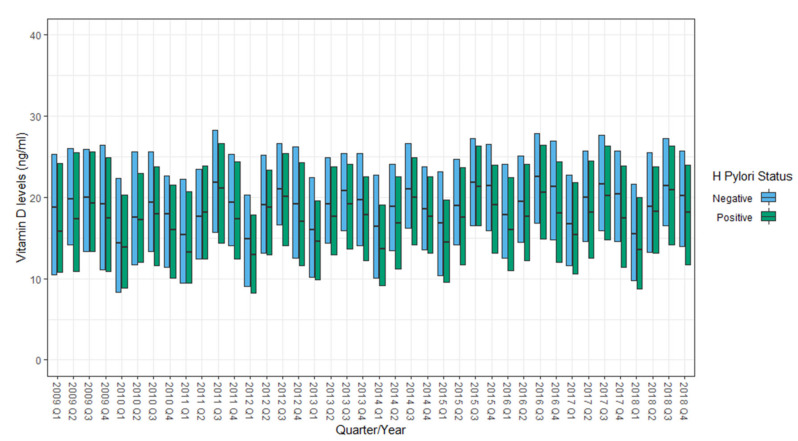
Distribution of serum vitamin D levels (median and IQR) by *Helicobacter pylori* status (positive or negative), yearly by quarter from 2009–2018.

Serum vitamin D levels were measured within a month of *H. pylori* testing in 27,077 (18%) subjects. Of these, 77% were performed within 10 days of the *H. pylori* testing. During the 10-year study period (2009 to 2018), serum vitamin D levels exhibited seasonal variability, being higher during warmer seasons (second and third quarters) and lower in the colder seasons (Figure 1). 

**Figure 2:**
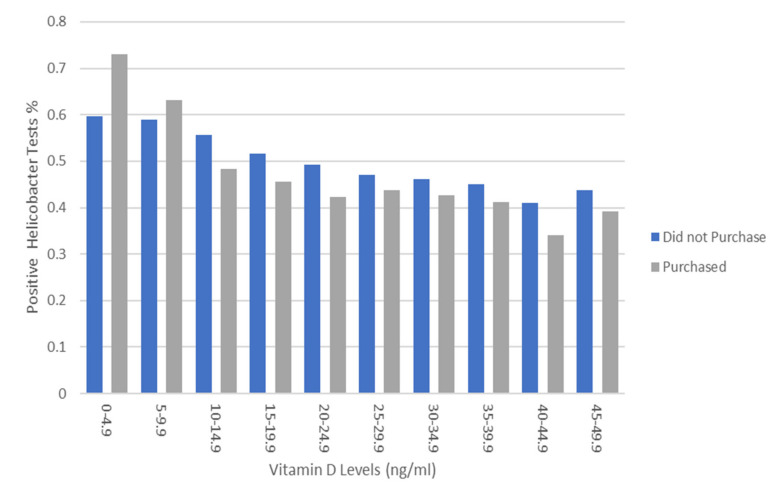
Prevalence of positive *H. pylori* tests by vitamin D level (ng/mL) and purchase history of vitamin D supplementation in the previous three months.

Mean serum vitamin D levels in *H. pylori* negative subjects were consistently higher than those observed in *H. pylori* positive subjects (20.1 ± 9.4 vs. 18.6 ± 9.8 ng/mL, *p*-value < 0.001) in 39 (out of 40) quarters of the study period (Figure 1). In a subgroup analysis of patients who were not prescribed PPIs in the three months prior to *H. pylori* testing (*n* = 153,314), *H. pylori* negative individuals had higher mean serum vitamin D levels than those who were *H. pylori* positive (19.96 ± 9.82 vs. 18.52 ± 9.4 ng/mL, *p*-value < 0.001). Starting from 1 April 2013, documentation of the purchase of prescription medications, OTC medications, and various dietary supplements (including vitamin D formulations) in all pharmacies affiliated with Meuhedet HMO became available for review. We ascertained 4227 subjects (4.9% of patients who underwent *H. pylori* testing after 1 April 2013) who also purchased various formulations of vitamin D within three months prior to *H. pylori* testing. Only 1184 of these patients underwent serum vitamin D level testing within 30 days of *H. pylori* testing. Mean serum vitamin D levels among subjects who purchased vitamin D supplementation were higher than those who had no record of such (21.97 ± 10.74 vs. 19.58 ± 9.32 ng/mL, *p*-value < 0.001). In a logistic regression analysis that controlled for gender, age, history of hypertension, diabetes mellitus, hypothyroidism, ischemic heart disease, and serum creatinine levels, for every 1 ng/mL increase of serum vitamin D, the odds of *H. pylori* infection decreased by 1.5% (OR = 0.985, 99% CI: 0.98–0.99, *p*-value < 0.001, McFadden-R^2^ = 0.83) (see Appendix A, Table A1). The odds of a positive *H. pylori* test were 31% higher among patients with serum vitamin D < 20 ng/mL, compared to those with vitamin D ≥ 20 ng/mL (OR 1.31, 99% CI 1.22–1.4, *p* < 0.001) (see Appendix B, Table A2). Moreover, a consistent decrease in the proportion of positive *H. pylori* tests was noted as serum vitamin D levels increased; this trend was observed both in patients who purchased vitamin D supplements and in those who did not (Figure 2).

**Figure 3:**
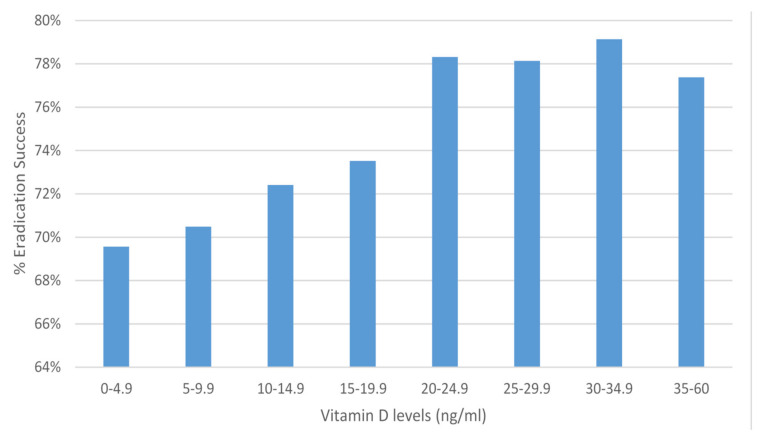
Percent of *Helicobacter pylori* eradication success, by vitamin D level (ng/mL).

In addition, in a logistic regression analysis that controlled for gender, age, history of hypertension, diabetes mellitus, hypothyroidism, ischemic heart disease, and serum creatinine levels, a significant inverse association between vitamin D purchasing (three months before *H. pylori* testing) and *H. pylori* infection was found (OR = 0.81, 99% CI: 0.72–0.91, *p* < 0.001, McFadden-R2 = 0.003) (see Appendix C, Table A3). Among *H. pylori* positive subjects, 61,921 underwent further *H. pylori* tests, which were considered to represent verification of eradication. Eradication of *H. pylori* was successful in 74% of the subjects. We ascertained 10,170 *H. pylori* positive subjects who had subsequent *H. pylori* testing as well as a measurement of serum vitamin D levels within thirty days. Mean serum vitamin D levels among those who succeeded in *H. pylori* eradication were found to be higher than in those who failed eradication (19.34 ± 9.55 vs. 18.64 ± 9.61, *p* < 0.001). A logistic regression analysis, controlling for patient’s age and appropriate anti *H. pylori* treatment, revealed that lower vitamin D levels (i.e., less than 20 ng/mL) were associated with *H. pylori* eradication failure with borderline significance (OR = 0.91, 99% CI: 0.8–1.0, *p* = 0.016, McFadden-R2 = 0.85) (Figure 3).

## 4. Discussion

The present study includes data from over 150,000 adult Israeli residents tested for the presence of *H. pylori* over a ten-year period. Our study discerned several notable associations between *H. pylori* infection, eradication, and serum vitamin D levels. Firstly, our results demonstrated that serum vitamin D levels were higher among *H. pylori* negative subjects, compared to the *H. pylori* positive subjects. Assuming a causal relation between vitamin D and *H. pylori*, the regression model (described in the results section) predicts that 5000–6000 out of the 100,000 cases of *H. pylori* infection could have been prevented by an increase in vitamin D from 15 to 30 ng/mL, independently of gender and history of hypertension, hypothyroidism, and serum creatinine levels. In addition, we demonstrated that the mean serum vitamin D levels among the 1184 subjects who purchased vitamin D supplementation were higher than those who had no record of vitamin D purchase. Moreover, the probability of a positive *H. pylori* test decreased as the serum vitamin D levels increased, regardless of vitamin D supplementation.

As many patients fail first line treatments of *H. pylori* [8], additional measures that can improve eradication rates are essential. Our study found that subjects who succeeded in *H. pylori* eradication had higher serum vitamin D levels compared to those who failed in *H. pylori* eradication, even after controlling for age and the type of antibiotic protocol given for *H. pylori* eradication. Assuming a causal relation between vitamin D and eradication of *H. pylori*, a person with vitamin D > 20 ng/mL has about a 2% higher likelihood of eradication than a person with vitamin D ≤ 20 ng/mL of the same age and using an identical antibiotic protocol. An association between low serum vitamin D levels and a high probability of *H. pylori* infection, as well as a lower eradication success rate of therapy of this pathogen, has been reported by others [9,10,11,12,13,14,15,16,17]. However, these studies included much smaller numbers of participants. The mechanisms underlying the association between low serum vitamin D levels and a *H. pylori* infection are unclear. It is known that *H. pylori* infection is associated with chronic gastritis. The presence of gastric inflammation may interfere with the absorption of iron and additional micronutrients [20]. It was reported that hypochlorhydria caused by *H. pylori*-related chronic gastritis causes a decrease in iron and vitamin B12 absorption [20]. Eradication of *H. pylori* was reported to heal the gastric inflammation, increase gastric acid secretion, and to improve iron and vitamin B12 absorption [20]. Data regarding the absorption of other micronutrients and vitamins like vitamin D, before and after *H. pylori* eradication, had not yet been published [20]. However, it was recently reported that hypochlorhydria may impair vitamin D absorption and may cause vitamin D deficiency [21]. Our findings raise the hypothesis that *H. pylori* negative subjects may have better absorption of vitamin D from their food intake, and thus exhibit higher serum levels of vitamin D, than subjects who are *H. pylori* positive. In our study, in addition to the subject’s serum vitamin D levels, we examined history of vitamin D acquisition to enhance causal inference regarding the association between vitamin D on *H. pylori* status and eradication success. We found a significant inverse association between vitamin D purchase and *H. pylori* infection. Similarly, researchers from Japan reported that long term administration of vitamin D for the treatment of osteoporosis in a small group of elderly women living in a nursing home was associated with a reduced rate of *H. pylori* infection [11]. The effect of vitamin D on *H. pylori* is supported by recently published basic research. Several antimicrobial effects against *H. pylori*, mediated by various metabolites or derivates of vitamin D, were described [22,23,24,25,26,27,28,29,30]. These include: (a) Alterations in the innate immune response: Administration of vitamin D upregulated the expressions of natural antimicrobial peptides like defensin β and cathelicidin [22,23,24]. These antimicrobial peptides may have a role in controlling *H. pylori* survival [25,26]. (b) Revitalization of the autolysosomal degradation function of the gastric epithelium against *H. pylori*: *H. pylori* invades the gastric epithelium where it becomes sequestered and survives in autophagosomes with impaired lysosomal acidification. Vitamin D3 treatment reactivates the lysosomal degradation function and cause the elimination of *H. pylori* [27]. (c) Bacterial cell wall lysis: It was recently reported that various vitamin D decomposition products interact with lipid components within the *H. pylori* membrane, destabilizing the membrane confirmation, and finally inducing a collapse of the cell membrane structure of *H. pylori*, ultimately causing bacterial cell wall lysis. These effects were found to be specific for *H. pylori* and were not found in other bacterial species [28,29,30]. Our study, to the best of our knowledge, is the largest study conducted to date to examine the association between serum vitamin D levels and *H. pylori*. Subjects in our study received their medical care in more than 600 different primary care clinics in different geographic regions in Israel. Thus, we can assume that our study population represents the general population. The EMRs provided a rich and comprehensive data source including all diagnoses and various laboratory and blood tests results, thus minimizing the problem of missing data, common in observational studies. There are some limitations to this study: Firstly, as an observational study, the available data were limited to that which exists in the EMRs. Neither the participants in this study nor their physicians could be directly queried about their sun exposure behaviors, purchase of medicine outside of the HMO-affiliated pharmacies, and indications for *H. pylori* and vitamin D levels testing. However, we may assume that the *H. pylori* testing was performed due to abdominal complaints and was not performed in asymptomatic subjects.

Secondly, we used an indirect measure of vitamin D supplementation. Assessment of vitamin D intake was based on purchase history of various formulations of vitamin D that was recorded in the patient’s EMR, in one of the multiple pharmacies affiliated with Meuhedet HMO distributed throughout the country. Although purchase history may be an indicator of drug exposure, it is not an accurate measure of drug adherence [31]. Thirdly, in this study, we had no information regarding the indication for vitamin D purchasing and the exact formulations of vitamin D (either vitamin D2 (ergocalciferol) or vitamin D3 (cholecalciferol) at different doses) that were purchased. It is known that the bioavailability of these products and the serum 25-hydroxyvitamin D levels after their ingestion may depend on several factors, including the type of vitamin D formulation that was ingested and the time that elapsed from the initiation of vitamin D ingestion [32]. Nonetheless, despite the missing information, the serum 25-hydroxyvitamin D levels in those who purchased any formulation of vitamin D were higher than those who did not have a history of vitamin D purchase. Fourthly, risk factors for acquiring *H. pylori* infection were not investigated in this study. Notably, those factors related to socio-economic status, which may be related to both vitamin D levels and *H. Pylori* infection, were not included in our data set. A literature review that summarized published data on the epidemiology of *H. pylori* in different populations suggested the following risk factors for *H. pylori* infection: male gender, increasing age, shorter height, tobacco use, obesity, and lower socioeconomic status [33]. As noted, Meuhedet HMO has a wide geographical distribution in the country; all ages were represented, including members from all socioeconomic groups; and the average income was slightly lower than the national average [34]. The prevalence of infection with *H. pylori* among our studied participants was more than 50%. Similar rates of *H. pylori* infection were reported from another HMO organization in Israel [35]. Analysis of the data from this HMO organization revealed that infection with *H. pylori* was significantly associated with the BMI of the studied participants (a cohort of 235,107 individuals aged 18 years and older). This association was significant after adjusting for age, sex, ethnicity, and socioeconomic status [35]. Another smaller study that involved Israeli children (Jewish children from the general population and of an ultraorthodox Jewish community and Arab children) disclose different prevalence of *H. pylori* infection among these communities. Data from this study revealed that socioeconomic factors may not totally explain the ethnic difference in *H. pylori* prevalence [36]. Fifthly, additional factors associated with low serum levels of vitamin D were not evaluated in this study. Despite being a sunny country, the prevalence of vitamin D deficiency or insufficiency in Israel is surprisingly high (more than 75%) [37]. A literature review that was published recently suggested the following predictors of low serum levels of vitamin D in subjects living in the Middle East and North Africa: female gender, increasing age, increasing BMI, religious dress code, winter season, use of sunscreens, and lower socioeconomic status [38], many of which could not be evaluated in this study.

This large scale EMR based study provides considerable data to support the relationship between *H. pylori* and vitamin D status. Several questions remain, including: the risk factors for acquiring *H. pylori* and for low vitamin D levels among Israeli subjects, the relationship between *H. pylori* related chronic gastritis and impaired absorption of vitamin D, and the relationship between *H. pylori* eradication and vitamin D absorption. Regarding therapeutics, relevant questions include the role of vitamin D supplementation to the general population in diminishing *H. pylori* infection rates; and whether vitamin D supplementation, in addition to standard antimicrobial therapeutics, would improve the eradication rates of *H. pylori*, especially of drug-resistant *H. pylori*. Prospective trials are needed to provide conclusive evidence for these latter questions.

## 5. Conclusions

Vitamin D levels are associated with *H. pylori* infection. Increased vitamin D levels are associated with successful *H. pylori* eradication. Vitamin D may have a role in *H. pylori* eradication.

## Figures and Tables

**Table 1:** Baseline demographic and clinical characteristics of the study population.

	H. pylori * Negative Subjects	H. pylori * Positive Subjects	*p*-Value	Effect Size
	(No. 74,843)	(No. 75,640)		
Age: Mean (SD), in years	42.15 (±16.65)	40.95 (±14.72)	<0.0001	−0.08
Females	50%	49%	<0.001	−0.02
BMI (kg/m^2^)	26.45 (±5.62)	26.89 (±5.64)	<0.0001	0.08
Smoking	0.16	0.22	<0.0001	0.15
Hypertension	0.08	0.06	<0.0001	−0.09
Type 2 Diabetes Mellitus	0.07	0.06	<0.0001	−0.04
Ischemic Heart Disease	0.04	0.03	<0.0001	−0.07
Congestive Heart Failure	0.01	0.01	<0.0001	−0.05
Atrial Fibrillation	0.02	0.01	<0.0001	−0.06
Crohn’s Disease	0.01	0.01	<0.0001	−0.06
Ulcerative Colitis	0.01	0.00	<0.0001	−0.04
Irritable Bowel Syndrome	0.10	0.07	<0.0001	−0.14
Liver Diseases (any type)	0.05	0.04	<0.0001	−0.06
Non-Alcoholic Fatty Liver Disease	0.05	0.04	<0.0001	−0.06
Hypothyroidism	0.09	0.07	<0.0001	−0.08
Osteoporosis	0.13	0.09	<0.0001	−0.11
Recent Omeprazole Use	0.03	0.02	<0.0001	−0.04
Recent Esomeprazole Use	0.04	0.02	<0.0001	−0.09

* *H. pylori*—*Helicobacter Pylori*.

## Data Availability

The data that support the findings of this study are available from Meuhedet Health Services. Restrictions apply to the availability of these data, which were used under license for this study. Data are available from the author with the permission of Meuhedet Health Services.

## Appendix A

Positive = 1

Negative = 0

Gender – M-Male

Call:

glm(formula = Positive_Or_Negative ~ VITAMIN_D + hypertension_diag +

  hypothyroidism_diag + Gender_Desc + creatinine, family = binomial,

  data = pat.unique)

Deviance Residuals:

 Min  1Q Median  3Q Max

−1.498 −1.196 1.033 1.142 2.536

Coefficients:

**Table A1:** Regression results for *Helicobacter Pylori* presence (*n* = 24,050), vitamin D as continuous variable.

	Estimate	Std. Error	z Value	Pr(>|z|)
Intercept	0.606679	0.060017	10.108	<2*10^−16^
VITAMIN D	−0.014985	0.001388	−10.796	<2*10^−16^
Prior Hypertension	−0.213496	0.051550	−4.142	3.45*10^−5^
Prior Hypothyriodism	−0.283841	0.048583	−5.842	5.15*10^−9^
Creatinine	−0.356375	0.077802	−4.581	4.64*10^−6^
Gender (male)	0.122431	0.031235	3.920	8.87*10^−5^

* as it is a symbol for level of significance which is stated.

(Dispersion parameter for binomial family taken to be 1)

  Null deviance: 33330 on 24049 df

Residual deviance: 33089 on 24044 df

AIC: 33101

Number of Fisher Scoring iterations: 4

## Appendix B

glm(formula = Positive_Or_Negative ~ low_vitd + hypertension_diag +

  hypothyroidism_diag + Gender_Desc + creatinine, family = binomial,

  data = pat.unique)

Deviance Residuals:

 Min  1Q Median  3Q Max

−1.432 −1.173 1.048 1.131 2.562

Coefficients:

**Table A2:** Regression results for *Helicobacter Pylori* presence (*n* = 24,050), vitamin D as dichotomous variable.

	Estimate	Std. Error	z Value	Pr(>|z|)
Intercept	0.18842	0.06137	3.070	0.00214
Vitamin D < 20 ng/mL	0.26957	0.02629	10.255	<2*10^−16^
Prior Hypertension	−0.21104	0.05154	−4.095	4.23*10^−5^
Prior Hypothyriodism	−0.28465	0.04856	−5.862	4.57*10^−9^
Creatinine	−0.38338	0.07782	−4.927	8.36*10^−7^
Gender (male)	0.12256	0.03126	3.921	8.81*10^−5^

* as a symbol for level of significance which is stated.

Signif. codes: 0 ‘***’ 0.001 ‘**’ 0.01 ‘*’ 0.05 ‘.’ 0.1 ‘ ’ 1

(Dispersion parameter for binomial family taken to be 1)

 Null deviance: 33330 on 24049 df

Residual deviance: 33102 on 24044 df

AIC: 33114

Number of Fisher Scoring iterations: 4

## Appendix C

Vitamin D Purchasing

glm(formula = Positive_Or_Negative ~ Vitamin_D_use_before + hypertension_diag +

  Ischemic_Heart_disease_diag + hypothyroidism_diag + creatinine,

  family = binomial, data = unique.purchase)

Deviance Residuals:

 Min  1Q Median  3Q Max

−1.351 −1.224 1.086 1.131 2.490

Coefficients:

**Table A3:** Regression results for *Helicobacter Pylori* presence (*n* =24,050), focusing on vitamin D purchase (dichotomous).

	Estimate	Std. Error	z Value	Pr(>|z|)
Intercept	0.39836	0.03906	10.200	<2*10^−16^
Prior VITAMIN D use	−0.20865	0.04562	−4.574	4.79*10^-6^
Prior Hypertension	−0.22597	0.03835	−5.892	3.83*10^−9^
Prior Ischemic Heart Disease	−0.19311	0.05221	−3.698	0.000217
Prior Hypothyriodism	−0.29300	0.03607	−8.124	4.53*10^−16^
Creatinine	−0.36114	0.04844	−7.455	9.00*10^−14^

* as it is a symbol for level of significance which is stated.

(Dispersion parameter for binomial family taken to be 1)

 Null deviance: 64764 on 46748 df

Residual deviance: 64505 on 46743 df

AIC: 64517

Number of Fisher Scoring iterations: 3
